# Mast Cells in Cardiac Remodeling: Focus on the Right Ventricle

**DOI:** 10.3390/jcdd11020054

**Published:** 2024-02-04

**Authors:** Argen Mamazhakypov, Abdirashit Maripov, Akpay S. Sarybaev, Ralph Theo Schermuly, Akylbek Sydykov

**Affiliations:** 1 Department of Internal Medicine, Excellence Cluster Cardio-Pulmonary Institute (CPI), Member of the German Center for Lung Research (DZL), Justus Liebig University of Giessen, 35392 Giessen, Germany; argen.mamazhakypov@innere.med.uni-giessen.de (A.M.); ralph.schermuly@innere.med.uni-giessen.de (R.T.S.); 2 Department of Mountain and Sleep Medicine and Pulmonary Hypertension, National Center of Cardiology and Internal Medicine, Bishkek 720040, Kyrgyzstan; ra.maripov@mail.ru (A.M.); ak_sar777@mail.ru (A.S.S.)

**Keywords:** mast cells, right ventricle, cardiac remodeling

## Abstract

In response to various stressors, cardiac chambers undergo structural remodeling. Long-term exposure of the right ventricle (RV) to pressure or volume overload leads to its maladaptive remodeling, associated with RV failure and increased mortality. While left ventricular adverse remodeling is well understood and therapeutic options are available or emerging, RV remodeling remains underexplored, and no specific therapies are currently available. Accumulating evidence implicates the role of mast cells in RV remodeling. Mast cells produce and release numerous inflammatory mediators, growth factors and proteases that can adversely affect cardiac cells, thus contributing to cardiac remodeling. Recent experimental findings suggest that mast cells might represent a potential therapeutic target. This review examines the role of mast cells in cardiac remodeling, with a specific focus on RV remodeling, and explores the potential efficacy of therapeutic interventions targeting mast cells to mitigate adverse RV remodeling.

## 1. Introduction

Cardiac remodeling refers to changes in size, mass, geometry, and function of heart, that develop in the course of various cardiovascular pathologies. It initially represents an adaptive process of the heart in response to mechanical, neurohumoral, or other stressors, with the primary aim of preserving cardiac function [1]. However, sustained exposure to pathological factors triggers the development of maladaptive cardiac remodeling. Cardiac remodeling can affect either the left or right ventricle (RV) or both ventricles and is associated with unfavorable outcomes and treatment responses [1,2]. It is now recognized as an important aspect of cardiovascular disease progression and is emerging as a therapeutic target in heart failure therapy [1]. 

Currently approved therapies have been efficient in reducing mortality and morbidity of left-heart failure patients [3,4,5]. Both pharmacological and non-pharmacological therapies beneficially impacted prognosis in heart failure patients by modulating the cardiac remodeling process [5,6,7]. Modulation of the renin-angiotensin-aldosterone (RAAS) and sympathetic nervous system is the cornerstone of pharmacotherapy of left heart failure [8]. Increased activity of the sympathetic nervous and RAAS has also been implicated in the pathophysiology for pressure overload-related RV failure [9,10,11]. Consequently, angiotensin-converting enzyme inhibitors, angiotensin II receptor antagonists, and β-blockers have demonstrated efficacy in reversing RV, remodeling in animal models [9,10]. However, currently, they are not recommended for therapy of RV failure associated with pulmonary hypertension due to inconsistent results in clinical trials [12]. It is important to note that most of the previous trials were small, underpowered, enrolled patients with systemic RV or predominantly focused on the pulmonary vasculature and did not assess RV remodeling [13,14]. In addition, the differences between the RV and left ventricle might account for the dissimilar responses of the failing ventricles to inhibitors of neurohormonal activity [15,16]. However, despite advances in treatment, the burden of heart failure remains high, emphasizing the ongoing need for further research and the development of novel management strategies [3]. 

Cardiac remodeling develops in response to stressors such as volume or pressure overload. Such unfavorable hemodynamic conditions occur in a variety of diseases, including hypertension, valvular pathologies, chronic pulmonary diseases, obesity, and metabolic disorders. To counteract the sustained rise in wall stress caused by excessive pressure and/or volume load, the myocardium undergoes phenotypic and functional transformations, which encompass a sequence of molecular, cellular, and interstitial alterations. Cardiomyocytes become enlarged due to new contractile protein synthesis, contributing to the increased size and mass of the affected cardiac chamber [17,18]. In addition to cardiomyocyte changes, cardiac remodeling involves alterations in other cell types and extracellular matrix organization. Fibroblast activation and proliferation result in the amplified synthesis of extracellular matrix proteins [19]. Changes in coronary microvascular endothelial cells lead to alterations in the coronary microvasculature and blood supply to the heart [20]. One of the important features of cardiac remodeling is the recruitment and accumulation of diverse inflammatory and immune cells within the myocardium [21,22], which can mediate both protective and deleterious effects [21]. 

## 2. Right Ventricular Remodeling

Recent studies have provided strong evidence to recognize the pivotal role of the RV in cardiovascular pathologies [23]. Functional and structural changes of the RV define the prognosis in patients with various cardiovascular diseases, including congenital heart disease [24], pulmonary arterial hypertension [25], myocardial infarction [26], advanced left heart failure [27] and stable coronary artery disease [28]. The recognition of the RV as a critical player in the progression of cardiovascular and respiratory conditions has sparked increased research attention towards the RV in recent years [29,30,31]. 

Pressure overload is a key pathogenetic factor for RV remodeling and dysfunction, which is associated with the release and subsequent accumulation of a myriad of bioactive molecules in the circulation and within the cardiac tissue [32,33]. These bioactive molecules have the potential to directly impose deleterious effects on the RV and modulate its response to pressure or volume overload [22]. 

Similar to the remodeling of the left ventricle, RV remodeling in response to pressure or volume overload is intricately associated with alterations in the function of cardiomyocytes, fibroblasts, endothelial cells, and various immune and inflammatory cells [34,35,36]. These cells are critically involved in cardiac remodeling, and their dysfunction can be caused by a multitude of factors and mediators synthesized and released by various cells within the RV myocardium [36]. Interactions between different cell types play a vital role in determining the overall outcome.

Another hallmark of RV remodeling is the increased production and deposition of extracellular matrix proteins. Ultimately, this alters the microenvironment of the myocardial cells and leads to a rise in tissue stiffness and expansion of the intercellular space within the heart [37]. Additionally, it creates a space for the build-up and storage of growth factors and inflammatory mediators.

The pathogenesis of the deleterious events during the course of RV remodeling involves a complex interplay of various processes and signaling pathways, including inflammation [22], extracellular matrix synthesis [38], calcium homeostasis [39], endothelial cell dysfunction [40], nitric oxide (NO) synthesis [41], endothelial-mesenchymal transition [42], matricellular protein synthesis [43] and growth factor signaling pathways such as transforming growth factor-β (TGF-β) [44] and apelin [45]. These interactions collectively dictate the fate of the RV under pressure or volume overload, and a better understanding of these intricate processes is essential to developing effective therapeutic strategies for the prevention or management of RV dysfunction and failure.

## 3. Mast Cell Biology

The pivotal role of mast cells in allergic responses has long been recognized [46]. However, recent studies have suggested their involvement in various pathological processes associated with different non-allergic diseases, such as tissue remodeling, repair, fibrosis, and angiogenesis [46,47]. Mast cells represent immune cells residing within the peripheral tissues and originate from bone marrow-derived precursor cells, regulated by key growth factors such as c-KIT ligand stem cell factor [48].

Mast cells express numerous cell surface antigens, including the cytokine receptor KIT (CD117) and the high-affinity receptor for the Fc region of immunoglobulin E (FcεRI). They are often identified as c-kit^+^FcεRI^+^ [49,50,51]. Mast cells also express the pan leukocyte antigen (CD45); however, they do not express surface antigens specific for other hematopoietic cells like CD2, CD3, CD4, CD11a, CD11b, CD11c, CD14, CD15, CD19, etc. [52,53]. Evaluation of the expression of these antigens allows distinguishing between mast cells and other CD45^+^ cells [54].

The most commonly used method for histochemical identification of mast cells in tissue sections is toluidine blue staining [55]. Morphologically, mast cells are identified by the presence of multiple large metachromatic granules in their cytoplasm, which store a variety of mediators, cytokines, and proteases [56]. 

**Figure 1:**
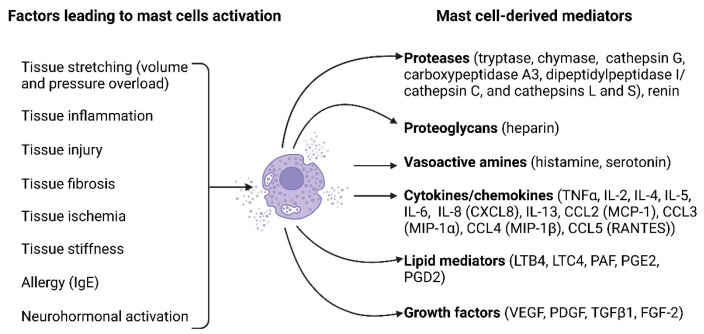
Mast cell activation factors and mast cell-derived mediators. A multitude of factors can induce mast cell activation and encompass tissue stretching due to pressure or volume overload, tissue inflammation, tissue injury, tissue fibrosis, tissue ischemia, tissue stiffness, allergies, and neurohormonal activation. Upon activation, mast cells release a diverse array of biologically active substances, including proteases (such as tryptase, chymase, cathepsin G, carboxypeptidase A3, dipeptidyl peptidase I (DPPI, cathepsin C), cathepsins L and S, and renin), proteoglycans (heparin), vasoactive amines (histamine and serotonin), cytokines/chemokines (such as TNFα, IL-2, IL-4, IL-5, IL-6, IL-8 [CXCL8], IL-13, CCL2 [MCP-1], CCL3 [MIP-1α], CCL4 [MIP-1β], and CCL5 [RANTES]), lipid mediators (LTB4, LTC4, PAF, PGE2, PGD2), and growth factors (VEGF, PDGF, TGFβ1, FGF-2). The figure was created using BioRender.com, accessed on 25 November 2023.

Mast cell activation can be mediated by the binding of antigens and antibodies to their membrane [57]. Additionally, activation may occur in response to diverse stimuli, including neuropeptides (substance P, vasoactive intestinal peptide), calcitonin gene-related peptide, and neurotensin), basic compounds (compound 48/80), inflammatory mediators, and certain drugs [57]. Figure 1 illustrates the key factors causing mast cell activation and the biologically active substances released by mast cells upon their activation.

Upon mast cell activation, their degranulation follows [58]. Degranulated mast cells in tissues can be identified using toluidine blue staining by a reduced granule content in their cytoplasm and by extracellular metachromatic granules in their immediate vicinity [59,60]. For the quantification of mast cell activation, the number of degranulated cells is expressed as a percent of the total number of mast cells [61]. Expression of some surface antigens, including CD63, CD200R1 and CD203c, was reported to significantly increase because of mast cell degranulation and was proposed as a surrogate molecular marker for mast cell activation [62]. However, each marker is probably related to a distinct activation mechanism [62].

Mast cells exert their effects through the release of various mediators, which are pre-stored in the granules or synthesized upon mast cell activation. The biologically active substances released by mast cells are then involved in various processes. Many factors released by mast cells, including histamine, heparin, proteoglycans, serotonin, and proteases, are preformed and stored in the granules [63]. In addition, mast cells synthesize a wide array of lipid-derived mediators, such as leukotrienes, prostaglandins, platelet-activating factor, and lipoxins [63]. They also produce various inflammatory cytokines like IL-1, IL-3, IL-4, IL-5, IL-6, IL-8, IL-10, IL-13, IL-16, and TNF-α, as well as growth factors like vascular endothelial growth factor (VEGF), basic fibroblast growth factor (bFGF), and transforming growth factor-beta (TGF-beta) [63]. Another group of bioactive factors released by mast cells are proteases, including tryptase, chymase, carboxypeptidase and cathepsin G [64,65]. The mast cell proteases are able to degrade extracellular matrix proteins such as collagen, elastin, and proteoglycans, activate matrix-degrading matrix metalloproteinases (MMPs), and are involved in the metabolism and composition of the extracellular matrix [64,65]. 

## 4. Mast Cell-Deficient Lines to Study Cardiac Physiology and Remodeling

Mast cell-deficient lines have significantly deepened our knowledge of mast cell biology and their role in diseases. The most frequently used in biomedical research mast cell-deficient mouse lines carry naturally occurring mutations in the white spotting (W) locus on mouse chromosome 5, which encodes the c-KIT proto-oncogene [66]. All these mutations lead to reduced receptor tyrosine kinase KIT-dependent signaling and severe mast cell deficiency, as the development of mast cells is critically dependent on the binding of the growth factor stem cell factor to its receptor KIT [67]. Furthermore, mutations at the mouse W locus cause defects in stem cells of the melanocytic, hematopoietic and germ cell lineages of various severity, which are not dependent on each other for their appearance [68,69]. Importantly, these disturbances in other cell types in mutant mice can influence the interpretation of mast cell-dependent phenomena.

Kit^W/W-v^ mice are double heterozygotes, which carry two mutant alleles, W and W^-v^ [70]. The W mutant allele is characterized by a point mutation at a splicing site in the transcript, resulting in a truncated KIT protein lacking kinase activity, whereas the W^v^ mutant allele represents a point mutation in the kinase domain of the c-KIT coding sequence, resulting in decreased tyrosine kinase activity [70]. Kit^W/W^ homozygotes mice display severe anemia and neutropenia; however, they die around the first week of life [68]. Kit^W-v^ homozygotes also exhibit severe anemia, and many of them survive to maturity (hence “v” for “viable”) [68]. Kit^W/W-v^ mice, which combine the severe Kit^W^ with the milder Kit^W-v^ mutation, have widely been used in biomedical research for their favorable survival and vigor [71].

Kit^W-sh/W-sh^ mice bear a spontaneous inversion mutation W-sash (W-sh) in the transcriptional regulatory elements upstream of the c-KIT transcription start site [72]. The defects caused by the W-sh mutations are confined to melanogenesis and mast cells. Heterozygotes are black with a broad white belt at the midline (hence the name and symbol sash) [73]. Kit^W-sh/W-sh^ mice are fertile and not anemic, which makes them more preferable than Kit^W/W-v^ mice in certain disease models [74]. However, the inversion in Kit^W-sh/W-sh^ mice also disrupts the gene encoding the pro-atrial natriuretic peptide (ANP) convertase corin, which is responsible for proteolytic cleavage of pro-ANP to produce ANP [75]. Consequently, Kit^W-sh/W-sh^ mice exhibit cardiomegaly [75] and poorly tolerate left ventricular pressure overload [75]. These findings suggest that, despite milder hematopoietic abnormalities, Kit^W-sh/W-sh^ mice might represent a less suitable line to study the role of mast cells in pressure overload-induced cardiac remodeling.

To differentiate the mast cell-specific contributions from the off-target effects of KIT-related defects in other cell types, reconstitution of these mice with wild-type bone marrow-derived and differentiated in vitro mast cells [74,76] has been utilized in various experimental models [77]. Furthermore, several Kit–independent mouse lines with more selective ablation of mast cells have been developed [77,78]. However, not all the Kit–independent mast cell-deficient mouse lines have been utilized to study the role of mast cells in cardiac remodeling. We have summarized in Table 1 studies that utilized mast cell-deficient lines to explore left and right ventricular remodeling.

Thus, while mast cell-deficient rodents have been instrumental in advancing our understanding of mast cell biology, their use in biomedical studies requires careful consideration of the specific research questions and potential confounding factors.

## 5. Mast Cells in Healthy Hearts

Besides cardiomyocytes, endothelial cells and fibroblasts, heart tissue contains a number of other cell types, including various immune and inflammatory cells [97,98]. The immune cells in the healthy myocardium comprise mast cells, macrophages, T lymphocytes (CD4^+^ and CD8^+^), natural killer cells, eosinophils, B cells, dendritic cells, neutrophils, monocytes, and plasma cells. These immune cells are involved in the maintenance of tissue homeostasis, regulation of inflammation, tissue repair, and heart protection against potential pathogens [98]. 

Cardiac mast cells are generally located in the interstitial space between cardiomyocytes and are closely associated with coronary vessels [99] and nerves [100]. They constitute only a small part of the immune cells present within the healthy myocardium. According to a recent single-cell RNA sequencing analysis, mast cells constituted less than 1% of all CD45^+^ immune cells in the healthy mouse myocardium [54]. In this study, the authors identified mast cells in cluster 19 of the single cell sequencing results, which expressed the mast cell marker Mcpt8 and the mast cell-associated cytokines IL4 and IL13 [54]. In the flow cytometry, the authors defined mast cells as CD45^+^CD11b^−^CD19^−^CD3e^−^ cells that were double positive for c-Kit (CD117) and FceR1 [54]. A relatively low abundance of mast cells in the myocardium was consistently demonstrated in histological studies in rodent and human heart tissues. In normal hearts, mast cell density ranges from 0.6 cells/mm^2^ in C57/BL/6 mice to 1.4 cells/mm^2^ in Wistar Kyoto rats [101] and to 6.8 cells/mm^2^ in dogs [102]. 

Mast cell distribution exhibits significant variation within the wall of mouse hearts; they are most prevalent in the epicardium (50%) and myocardium (45%), with fewer numbers in the endocardium (5%) [103]. In contrast, in healthy rats, mast cell numbers appear to be similar in both the subepicardial and subendocardial layers of the left ventricle [104]. 

The existing evidence suggests variations in mast cell density between the right ventricle and left ventricle in both healthy and diseased hearts. Assessment of mast cell density in healthy hearts across various species used in animal models, including rats, mice, and dogs, produced somewhat inconsistent results (Table 1). In rats, mast cell density was reported in some studies to be higher in the left ventricle [105,106], while others found higher mast cell numbers in the right ventricle [107] (Table 1). In healthy mouse hearts, mast cell density is higher in the RV compared to the left ventricle and interventricular septum [103]. No differences in mast cell density between the ventricles were reported in dogs [108] (Table 2). Furthermore, aging in rats is associated with an increase in mast cell density within the myocardium of both ventricles [105].

Mast cells are known for their heterogeneity, so the phenotypes of cardiac mast cells differ from those found in other tissues. In contrast to mast cells found in human skin and lungs, mast cells in human hearts demonstrate slightly different patterns of mediator release and synthesis [113]. Upon cross-linking of IgE receptors on human heart mast cells, histamine, tryptase, leukotriene C4, and prostaglandin D2 are released, although they differ quantitatively and qualitatively compared to skin and lung mast cells [113]. Mast cell heterogeneity is also evident from the patterns of mediator release. Human heart mast cells respond to C5a and protamine by releasing histamine in a similar way as skin mast cells; however, they differ from lung mast cells in this aspect [113]. Further, human heart mast cells do not respond to substances P and morphine, which activate skin mast cells [113]. Overall, the unique phenotypes and response patterns of cardiac mast cells in comparison to mast cells in other tissues suggest that these cells might play specific roles in cardiac physiology and immune responses within the heart. 

Increasing evidence suggests that cardiac mast cells might play a role in physiological conditions in the absence of injury [114]. Differences in cardiac structure and function between healthy mast cell-competent and mast cell-deficient rats at different ages suggest that mast cells may be an important factor in maintaining myocardial homeostasis in healthy rats [115]. 

## 6. Mast Cells in Right Ventricular Physiology and Remodeling

Various immune and inflammatory cells are crucially involved in cardiac remodeling [22]. Notably, the content of immune cells in the RV differs from that in the left ventricle in physiological as well as pathological conditions [116]. Specifically, the number of CD45^+^/CD11b/c^+^ cells was on average four times higher in the RV than in the left ventricle in rats exposed to either normoxia or hypoxia, suggesting a higher content of immune cells in the RV. A substantial body of evidence has accumulated supporting the involvement of mast cells in left heart hypertrophy and failure. However, the role of mast cells in RV hypertrophy and failure remains insufficiently explored.

In pathological conditions associated with left ventricular remodeling, mast cell numbers in the left ventricle were increased and exhibited more dynamic changes compared to the RV in animals as well as in humans (Table 1). In a rat model of myocardial infarction, mast cell density in the left ventricular infarct region peaked at 1 day and 3 weeks post-infarction, while RV mast cell density remained unchanged [110]. Similarly, in spontaneously hypertensive rats (SHR), the extent of mast cell accumulation over time was greater in the left ventricle than in the RV [106,111]. Thus, the left ventricular myocardium appears to inherently possess a higher number of tissue mast cells than the RV in both health and disease conditions (Table 1). However, it is essential to note that most of these studies focused on the left ventricle, and currently, there are no studies evaluating changes in mast cell distribution in the left ventricle in models of RV failure. The functional implications of the distinct mast cell pools between the ventricles remain to be fully understood.

**Figure 2:**
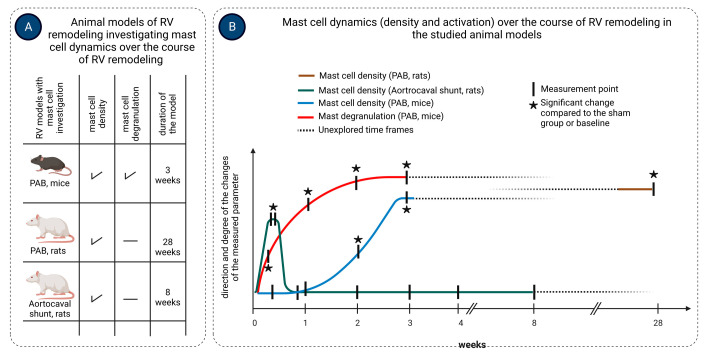
Mast cell density and activation in experimental models of right ventricular (RV) remodeling. Mast cell density and activation were studied in mice and rats subjected to pulmonary artery banding (PAB) for various times up to 3 weeks and 28 weeks, respectively, and in rats subjected to aortocaval shunt for 8 weeks (**A**). Mast cell density was assessed in the RV tissue in all three models, while mast cell activation (degranulation) was evaluated only in the PAB mouse model. Mast cell density increased significantly in the RV of PAB mice and rats, starting at week 2 and remained elevated up to the end of the observation (**B**). In contrast, mast cell density in the RV of rats subjected to aortocaval shunt increased early during the first few days after surgery, returned to baseline values within the first week, and remained at these low values over the course of 8 weeks (**B**). Activation of mast cells (degranulation) in the RV of PAB mice occurred as early as three days after PAB surgery and persisted over the course of 3 weeks. Arrows indicate the time points of the measurements. The figure was created using BioRender.com, accessed on 25 November 2023.

Activation of mast cells in response to pressure overload occurs early after exposure to the stressor, as evidenced by increased mast cell degranulation in the RV of mice 3 days following pulmonary artery banding (PAB) surgery [117]. The timing of mast cell accumulation during RV remodeling might depend on the type of overload and is summarized in Figure 2. In a rat aortocaval fistula model of volume overload, mast cell density in the RV significantly increased in the first days after surgery, returning to normal values on the third day and not changing thereafter over 56 days [118], suggesting an important role of mast cells in the initial stages of volume overload-induced RV remodeling. On the contrary, in mice subjected to PAB, RV mast cell density significantly increased two weeks after induction of the pressure overload and remained elevated three weeks post-surgery [117]. Observations in PAB rats showed that mast cell density in the RV was significantly increased as late as 200 days following pressure overload induction [119]. 

The source of increased mast cell numbers in RV remodeling remains to be elucidated. In left ventricular remodeling induced by angiotensin II infusion, the increase in mast cell density was shown to result from the maturation of pre-existing immature mast cells [89]. Similarly, resident mast cells within the RV myocardium might undergo proliferation and maturation during RV remodeling. Alternatively, circulating mast cell progenitors might be recruited to the RV myocardium in response to volume or pressure overload. 

SHR commonly serves as a model of systemic arterial hypertension and left ventricular hypertrophy. Enhanced myocardial fibrosis, increased collagen volume fraction, and elevated hydroxyproline levels in the left ventricle in SHR were closely linked to an increased accumulation of mast cells in the left ventricle [106]. Interestingly, a modest degree of myocardial fibrosis and mast cell accumulation were also observed in the RV of SHR, which might be accounted for by pulmonary hypertension [120,121] probably secondary to left heart disease. It should be noted that the extent of pathological changes and mast cell accumulation in the RV of SHR was minor compared to the left ventricle, which was likely due to differences in the magnitude of pressure load exerted on each ventricle [106]. The involvement of mast cells in RV remodeling is corroborated by findings of a progressive accumulation of mast cells in RV in SHR in the advanced stages of the disease, which is associated with the development of RV hypertrophy and fibrosis [111].

The evidence for the pathological role of mast cells in adverse RV remodeling was further provided in experiments exploiting mast cell-deficient mice and pharmacological inhibition of mast cell activation [87]. Treatment of wild-type mice subjected to pressure overload by PAB with the mast cell stabilizer cromolyn significantly improved RV remodeling and function [87]. Similarly, mast cell-deficient Kit^W^/Kit^W-v^ mice subjected to PAB showed minimal RV dilation and preserved RV function [87]. These beneficial changes in RV remodeling were associated with reduced gene expressions in the RV of proinflammatory cytokines, such as TNF-α and IL-6 [87]. Notably, both TNF-α and IL-6 were previously shown to be involved in pressure overload-induced left ventricular remodeling [122,123] and mast cells were found to be the main source of IL-6 in pressure-overloaded myocardium [54]. 

Accumulating evidence suggests that mast cells may also play a protective role in myocardial remodeling [51,124]. Moreover, mast cells and their granules exert protective effects in the acute phase after myocardial infarction in rats via improving cardiomyocyte survival and vascularization [110]. Mast cell population heterogeneity could account for the diversity of mast cell effects [125]. Furthermore, mast cells release a wide range of mediators with multiple potential functions, which can exert opposite effects, so that the net effect might vary at different disease stages and in various disease conditions depending on the predominant involvement of individual mediators [126]. For instance, mast cell-derived histamine can inhibit renin release by mast cells in cardiac ischemia-reperfusion via activation of H4 receptors on the mast cell membrane and provide cardioprotection [127]. On the contrary, adenosine triphosphate released by mast cells during cardiac ischemia-reperfusion can amplify renin release from mast cells by positive feedback in an autocrine mode and worsen cardiac dysfunction [128].

This dual functional role of mast cells in myocardial remodeling can be nicely illustrated by the switch in phenotype and role of mast cells in failing human hearts caused by the change in disease conditions [129]. Particularly, myocardial tissues from patients with dilated and ischemic cardiomyopathies were characterized by enhanced accumulation of chymase-positive mast cells, which were localized in areas of enhanced interstitial fibrosis [129]. Diminished fibrosis following a long-term mechanical unloading of the hearts was associated with a secondary elevation in the myocardium of predominantly chymase-negative mast cells and decreased myocardial bFGF levels [129].

## 7. Mechanisms of Mast Cell-Mediated Effects on the Heart

### 7.1. Effects of Mast Cells on Cardiomyocytes

Mediators released by activated mast cells may exert direct effects on cardiomyocytes and impact their survival, apoptosis, contraction, and arrhythmogenesis. Experiments with adoptive transfer of bone marrow-derived mast cells from wild-type mice or corresponding knockout mice to mast cell-deficient Kit^W-sh/W-sh^ mice with diabetic cardiomyopathy demonstrated a role of mast cell-derived IL6 and TNF-α in promoting cardiomyocyte death [130]. The mast cell-derived mediator histamine, which is present in the human heart at high concentrations, was shown to reduce cell viability and induce cardiomyocyte apoptosis [131]. In line with these observations, pharmacological inhibition, or genetic disruption of the histamine H2 receptor slowed heart failure progression in mice subjected to pressure overload through a reduction of myocardial apoptosis [131]. In vitro experiments confirmed that activation of histamine H2 receptors increases apoptosis in isolated neonatal rat cardiomyocytes [131]. Mast cell proteases can induce cardiomyocyte apoptosis through other mechanisms [95,132]. Thus, mouse mast cell protease-4 promoted cardiomyocyte apoptosis via the degradation of insulin-like growth factor-1, which serves as a survival factor for cardiomyocytes [95].

In cardiac volume overload, chymase uptake by cardiomyocytes resulted in myosin degradation and cardiomyocyte dysfunction [133]. In contrast, mast cell deficiency led to reduced contractility and myofilament Ca^2+^ sensitization after myocardial infarction in mice, suggesting an important role of cardiac mast cells in preserving postischemic cardiac function [51]. 

Evidence suggests that cardiac mast cells can modulate cardiac electrical activity. Non-specific activation of resident mast cells by secretagoges affected the ability of pacemaker cardiomyocytes to generate spontaneous action potentials in the sinoatrial node and caused a shift in the activation pattern [134]. 

Mast cells are the main source of renin in both human and rodent myocardium [100,135]. Renin derived from mast cells in ischemia/reperfusion activated cardiac RAAS and caused excessive norepinephrine release, leading to arrhythmias [135]. Concordantly, suppression of renin release by activation of Gi-coupled receptors and retinoic acid receptors on mast cells inhibited local RAAS and provided cardioprotection [127,136,137]. Furthermore, mast cell-deficient mouse hearts exhibited reduced renin release during reperfusion and were markedly protected from ischemia-reperfusion-induced arrhythmias [135]. 

### 7.2. Mast Cells and Extracellular Matrix Modulation

Myocardial fibrosis plays a pivotal role in the pathogenesis and progression of various cardiac diseases [38]. Elucidating the precise mechanisms underlying myocardial fibrosis development is crucial for understanding disease pathogenesis and identifying novel therapeutic targets [138]. Myocardial fibrosis involves an interplay of versatile processes, including the synthesis and turnover of extracellular matrix proteins, the activation and proliferation of cardiac fibroblasts, as well as the synthesis of diverse inflammatory mediators and growth factors with potent pro-fibrotic properties [139]. These intricate events are meticulously orchestrated by various cell types residing within the myocardium, including various immune cells [139,140,141]. 

Recent studies have implicated mast cells as significant contributors to myocardial fibrosis [142]. In patients with idiopathic dilated cardiomyopathy, a notable association was demonstrated between mast cell density and the severity of myocardial fibrosis [143]. Similarly, mast cell density is positively correlated with the percentage of collagen fibers in the left ventricle in hypertensive heart disease patients [144]. Remarkably, mast cells were localized near areas of myocardial fibrosis in end-stage heart failure patients [129]. Co-culture of healthy fibroblasts with mast cells isolated from cardiac tissues of heart failure patients increased their collagen production [145], further supporting the potential role of mast cells in myocardial fibrogenesis. Accordingly, a reduction in myocardial fibrosis after long-term left ventricular assist device support (>40 days) was associated with a decreased ratio of chymase-positive to total mast cell numbers [129]. 

Experimental studies provided further evidence to support the link between mast cell activation and myocardial fibrosis development. In Dahl salt-sensitive rats, degranulated mast cells were found to localize near fibrotic regions in the myocardium [146]. Notably, mast cells underwent degranulation during the progression from heart hypertrophy to failure in those rats by releasing pro-fibrotic mediators and inducing augmented myocardial fibrosis [146]. Activation of the εPKC signaling pathway in mast cells was responsible for mast cell degranulation and subsequent TGF-β release [146]. In line with these findings, in spontaneously hypertensive rats, expression of mast cell-derived TGF-β and bFGF significantly increased during the transition from cardiac hypertrophy to heart failure and was associated with exaggerated myocardial fibrosis [147]. 

Chymase inhibition with chymostatin reduced myocardial active TGF-β1 levels in rats subjected to pressure overload by transverse aortic constriction (TAC), suggesting that activation of latent TGF-β1 is one of the pathways by which cardiac mast cell-derived chymase contributes to myocardial fibrosis [148]. Besides TGF-β1 activation, chymase induced TGF-β1 expression in a dose-dependent fashion in rat cardiac fibroblasts [149]. Further experiments confirmed that mast cell-derived chymase promotes cardiac fibroblast proliferation and collagen synthesis via the TGF-β/Smad pathway [149]. Importantly, chymase inhibition significantly suppressed cardiac fibrosis in cardiomyopathic hamsters [150]. Chymase expression in left ventricular tissue was correlated with increased mast cell density in terminal heart failure patients [151], suggesting that mast cell-derived chymase in heart failure might represent a potential therapeutic target. 

Another pathway by which mast cells can contribute to myocardial fibrosis is the activation of the RAAS in the heart. Mast cells are recognized as a significant source of renin in the myocardium, which consequently converts angiotensinogen to angiotensin I [100,152]. Next, cathepsin G [153] and chymase [154,155] derived from activated mast cells convert angiotensin I into angiotensin II. Angiotensin II subsequently exerts pro-fibrotic effects on the myocardium [156] through angiotensin II receptors, which are present on cardiac fibroblasts and mediate multiple pro-fibrotic effects [156,157]. 

Activated mast cells release a number of other factors, including histamine [131] and tryptase [101,158] that might account for their pro-fibrotic activities on cardiac fibroblasts. Treatment of isolated cardiac fibroblasts with tryptase induced increased collagen synthesis and fibroblast proliferation [101,158]. Collagen production was enhanced via activation of the protease-activated receptor-2 and ERK1/2 signaling [158]. The relevance of in vitro findings was further corroborated by the reduction of myocardial fibrosis in spontaneously hypertensive rats treated with a tryptase inhibitor [158]. 

**Figure 3:**
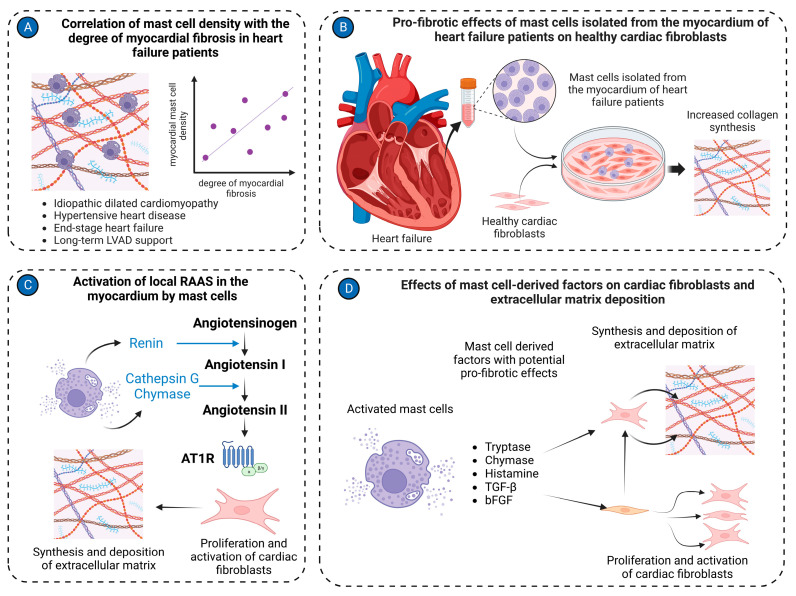
Mast cell in cardiac fibrosis. (**A**) Mast cell density positively correlates with the degree of myocardial fibrosis in the left ventricular tissues isolated from patients with various cardiac pathologies, including idiopathic dilated cardiomyopathy, hypertensive heart disease, end-stage heart failure, and long-term LVAD support; (**B**) Mast cells isolated from cardiac tissues of heart failure patients stimulate activation of healthy cardiac fibroblasts, causing them to synthesize and release extracellular matrix molecules in co-culture experiments; (**C**) Mast cells activate the local RAAS in the myocardium. Renin produced by mast cells can convert angiotensinogen to angiotensin I. Cathepsin G and chymase derived from mast cells transform angiotensin I to angiotensin II. Angiotensin II stimulates cardiac fibroblasts to synthesize extracellular matrix molecules; (**D**) Several factors derived from mast cells, such as tryptase, chymase, histamine, TGF-β, and bFGF, activate cardiac fibroblasts to myofibroblasts and stimulate production of extracellular matrix molecules. The figure was created using BioRender.com, accessed on 25 November 2023.

MMP/tissue inhibitors of the metalloproteinases system play a key role in the modulation of the extracellular matrix in pathological conditions. Several lines of evidence implicate mast cells in the regulation of this system in volume overload-induced cardiac remodeling (Figure 3). A strong correlation was reported between mast cell density and MMP activity in the hearts of rats subjected to volume overload [118]. Application of the secretagogue compound 48/80 [159] or endothelin-1 [160] in isolated rat hearts caused cardiac mast cell degranulation and activation of MMPs, leading to collagen degradation and moderate ventricular dilation. Furthermore, in a model of volume overload induced by aortocaval fistula, which encodes a mast cell secretagogue substance P and neurokinin A, mice lacking the TAC1 gene were protected from adverse left ventricular remodeling associated with attenuated myocardial MMP activity and collagen degradation due to reduced mast cell degranulation [161]. Mast cell activation, along with enhanced MMP activity and increased collagen degradation, was also reported in a model of dyslipidemia-associated dilated cardiomyopathy induced by a high-fat diet in ApoE^−/−^ mice [162]. Co-culture experiments further supported the role of mast cells in increasing MMP-2 activity and expression in fibroblasts [163]. The importance of mast cells in mediating MMP activation and collagen degradation is emphasized by findings of attenuated MMP-2 activity and ameliorated left ventricular dilation in mast cell-deficient rats subjected to aortocaval fistula-induced volume overload [164]. 

### 7.3. Mast Cells and Myocardial Vascularization

**Figure 4:**
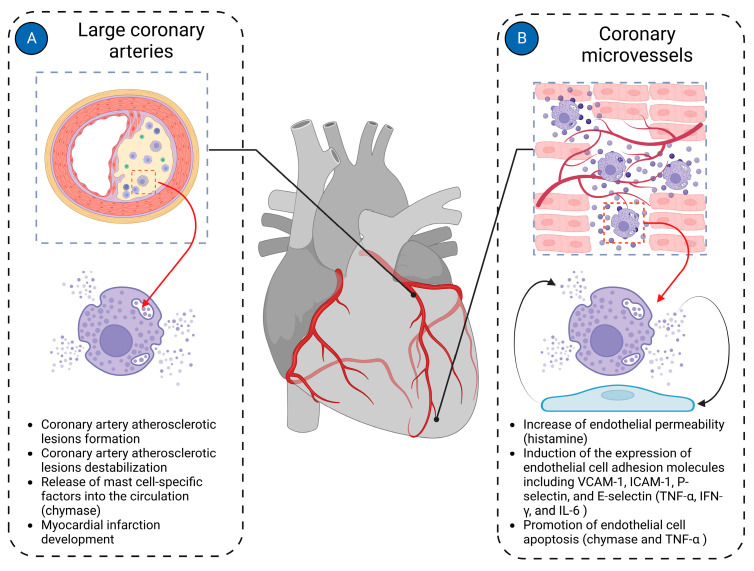
Mast cells in cardiac vasculature. Mast cells in the cardiac vasculature have been implicated in the pathologies of both large coronary arteries and coronary microvasculature. (**A**) In large coronary arteries, mast cells are mainly involved in the pathogenesis of coronary atherosclerotic plaque formation and contribute to its instability, ultimately leading to the development of myocardial infarction; (**B**) In coronary microvessels, mast cells primarily interact with endothelial cells, modulating their functions. Histamine derived from mast cells increases the permeability of endothelial cells. Mast cell-derived TNF-α, IFN-γ, and IL-6 induce expression of endothelial cell adhesion molecules such as VCAM-1, ICAM-1, P-selectin, and E-selectin. Chymase and TNF-α derived from mast cells promote endothelial cell apoptosis. The figure was created using BioRender.com, accessed on 25 November 2023.

Most cardiac pathologies are associated with changes in myocardial vascularization [40,165]. Mast cells are present in the walls of both microvessels and larger coronary arteries, suggesting their potential involvement in both micro- and macrovascular pathologies of the coronary arteries (Figure 4). 

Mast cells have been shown to induce left ventricular diastolic dysfunction in various animal models of heart failure [166,167]. Abnormal mast cell activation was identified as a cause of cardiac microvessel disease in Lepr^db/db^ female mice, another experimental model of diastolic dysfunction associated with heart failure with preserved ejection fraction [61]. The cardiac microvessel disease was characterized by enhanced vessel permeability due to disruption of endothelial adherens junctions by mast cell-derived histamine [61]. Histamine released by mast cells may interact with both H1 and H2 receptors to exert its effects on the cardiac endothelial cells and contribute to heart failure development [168]. Notably, the use of H2 receptor antagonists in the aging human population was associated with reduced risk for incident heart failure and favorable effects on the heart [169].

Increased serum tryptase levels were reported in patients with stable coronary artery disease, suggesting that chronic low-grade inflammation in atherosclerotic plaques triggers mast cell activation [170]. In vitro experiments demonstrated that activated mast cells might contribute to plaque erosion by inducing endothelial cell apoptosis [171,172]. Indeed, recent evidence implicates mast cells in plaque destabilization and atherosclerotic coronary complications. Mast cell density increased from 0 in controls to 2.3–2.7 per mm^2^ in the media layer of the coronary artery, with the highest numbers observed in unstable plaques of myocardial infarction patients [173]. In patients with myocardial infarction, segments with plaque rupture demonstrated significantly higher numbers of adventitial mast cells compared to segments with non-ruptured plaques or normal intima of the infarct-related coronary artery [174]. 

In contrast to the abovementioned reports, accumulating evidence suggests that mast cells exert beneficial effects on maintaining cardiac microvascular homeostasis. In left anterior descending artery (LAD) occlusion experiments, implantation of wild-type mast cells into mast cell-deficient c-Kit Kit^W/W-v^ mice enhanced angiogenesis, improved cardiac function, and decreased infarct size at early time points after myocardial infarction [84]. Indeed, in several in vitro studies, mast cells promoted angiogenesis by stimulating endothelial cells to release angiogenic factors [175,176]. 

### 7.4. Mast Cells and Myocardial Inflammation

Myocardial inflammation in various cardiac pathologies is recognized as a significant contributor to adverse myocardial remodeling in both ventricles [22]. The importance of mast cells in this process is emphasized by the fact that they possess a variety of inflammatory mediators in their granules and are able to rapidly generate more inflammatory agents upon activation [177]. Activated mast cells release a number of inflammatory factors, including interleukins (IL-1, IL-3, IL-4, IL-5, IL-6, IL-8, IL-10, IL-13, and IL-17), TNF-α, and chemokines (CXCL8/IL-8, CCL2/MCP-1, CCL3/MIP-1α, CCL4/MIP-1β, and CCL5/RANTES) [56]. 

Inflammatory mediators released by mast cells can enhance myocardial inflammation by activating further inflammatory processes. In canine experimental cardiac ischemia-reperfusion, mast cells primarily released TNF-α, which then exacerbated myocardial inflammation and cardiac injury by upregulating IL-6 in infiltrating leukocytes and initiating the cytokine cascade [178]. In line with this report, attenuated myocardial injury in mast cell-deficient Kit^W^/Kit^W-v^ mice following myocardial ischemia-reperfusion was associated with lower serum IL-6 compared to their wild-type counterparts [83]. Importantly, mast cell stabilization with ketotifen and cromolyn sodium prevented an increase in myocardial TNF-α levels following reperfusion [179]. 

Markedly elevated myocardial TNF-α levels in response to cardiac volume overload were observed in wild-type rats at 5 days post-fistula; conversely, TNF-α was almost undetectable in the hearts of mast cell-deficient rats [164]. In a similar model, protection from adverse left ventricular remodeling in mice lacking the TAC1 gene was associated with reduced mast cell degranulation and attenuated TNF-α expression [161]. 

Growing evidence suggests that inflammatory mediators released from mast cells are critically involved in the development of pressure overload-induced cardiac remodeling. In spontaneously hypertensive rats, left ventricular nuclear factor kappa-B and IL-6 expression in mast cells were already increased during the prehypertensive stages [147]. Stimulation of cardiac mast cell degranulation with the compound 48/80 in an ex vivo Langendorff heart preparation resulted in increased expression of nuclear factor kappa-B and IL-6 mRNA in the left ventricles [147]. These data are supported by recent findings of single-cell sequencing of immune infiltrates in the left ventricles of mice subjected to pressure overload, which revealed that mast cells had the highest expression of IL-6 among all immune cells [54]. 

Mast cell-derived inflammatory mediators have recently been implicated in the pathogenesis of cardiometabolic diseases. Streptozotocin-induced diabetic cardiomyopathy in mice is characterized by pathologic myocardial inflammation and the accumulation of mast cells in the heart [130]. Remarkably, mast cell-deficient Kit^W-sh/W-sh^ mice were protected from diabetic cardiomyopathy [130]. Preserved cardioprotection following adoptive transfer of bone marrow-derived mast cells from Tnf-deficient but not wild-type mice into Kit^W-sh/W-sh^ mice treated with streptozotocin identified TNF-α released by mast cells as the key mediator in this pathology [130].

## 8. Mast Cells as a Therapeutic Target

Preclinical studies have provided evidence that mast cells may represent an attractive target in the prevention and treatment of heart diseases. Treatment approaches in the context of heart failure may involve targeting different stages of mast cell development and activation, including blocking the processes that activate mast cells, targeting specific mast cell-derived mediators and their receptors on cardiac cells, limiting mast cell proliferation, or promoting mast cell apoptosis [180]. Some of these strategies have been explored in rodent models of heart failure. 

Prevention of mast cell activation by stabilization of their membranes has demonstrated efficacy in improving myocardial remodeling in various experimental models of heart failure. In SHR, mast cell stabilization with nedocromil prevented left ventricular fibrosis, inflammatory cell recruitment, and cytokine overexpression in the myocardium without affecting blood pressure or left ventricular hypertrophy [101]. Mast cell stabilization with cromolyn sodium attenuated left ventricular remodeling and left ventricular diastolic dysfunction in ovariectomized Fischer rats [166], ameliorated left ventricular diastolic dysfunction in Lepr^db/db^ female mice [61], and attenuated RV dilatation and improved RV function in mice subjected to PAB [87]. The class effects of these drugs were further supported by the prevention of the transition from compensated hypertrophy to heart failure in animals subjected to abdominal aortic banding using another mast cell stabilizing agent, tranilast [85]. 

Many currently approved pulmonary arterial hypertension therapies represent repurposed drugs, and drug repurposing continues to be an attractive approach to developing novel PAH therapies [181]. One of the advantages of already approved drugs is that they have a well-established safety profile [182]. This helps mitigate the costs and time associated with novel drug development [182]. Cromolyn sodium is safe and has been approved for treatment of mastocytosis and allergic diseases, such as bronchial asthma, conjunctivitis, rhinitis, etc. Therefore, cromolyn represents a promising candidate for clinical development for the treatment of RV failure in humans.

Mast cell-derived chymase has been identified as one of the key mast cell-specific targets in various cardiac pathologies [183]. Chymase inhibition improved cardiac remodeling in various animal models, including rapid ventricular pacing-induced heart failure in dogs [155], LAD-occlusion-induced myocardial infarction in rats [184] and hamsters [185], ischemia-reperfusion cardiac remodeling in pigs [186], and pressure overload-induced left ventricular remodeling in rats [148]. Further, mice lacking mMCP-4, the mouse counterpart of human mast cell chymase, were protected from adverse cardiac remodeling and dysfunction in the LAD-occlusion model of myocardial infarction [94,95,96]. Mast cell chymase can limit the cardiac efficacy of the angiotensin-converting enzyme (ACE) inhibitor therapy in rodents [187]. Therefore, combined chymase and ACE inhibition, compared to ACE inhibition alone, achieved better results in left ventricular function improvement, amelioration of adverse cardiac remodeling, and improvement of survival after myocardial infarction in hamsters [187]. The role of chymase inhibition in RV remodeling and dysfunction, however, remains unexplored, warranting future studies. 

Blocking receptors for mast cell-derived mediators such as histamine H2 receptor improved cardiac function in TAC mice [131] and in dogs with pacemaker-driven tachycardia [188]. Interestingly, a large prospective observational cohort study of participants without cardiovascular disease at baseline showed that baseline use of H2 receptor antagonists was associated with a 62% lower risk of incident heart failure [169]. The beneficial effects of H2 receptor antagonists on all-cause mortality in patients with different clinical forms of pulmonary hypertension suggest that these drugs exert direct effects on the right ventricle [189]. In line with these observations, H2 receptor antagonist use in the general population was associated with lower RV mass and smaller RV end-diastolic volume [190]. A meta-analysis revealed that H2 receptor antagonists may improve cardiac function in heart failure patients by decreasing myocardial oxygen demand due to negative inotropic and chronotropic effects [191]. It should be noted that H2 receptors are present not only in cardiac cells but also in various blood cells, such as leukocytes, macrophages, neutrophils, thrombocytes, and erythrocytes [168]. Consequently, the indirect effects of other cells expressing H2 receptor might modulate the effects of H2 receptors antagonists on the heart. 

## 9. Conclusions and Future Directions

In this comprehensive review, we have conducted a thorough examination of the existing literature to elucidate the role played by mast cells in the initiation and progression of myocardial remodeling, with a particular focus on dissecting mast cell involvement in RV remodeling and dysfunction. 

Mast cells are the major source of a whole host of biologically active substances, including growth factors, proteases, cytokines, chemokines, polypeptides, biogenic amines, proteoglycans, and phospholipid metabolites [192]. The biological complexities of mast cells in the setting of cardiac remodeling are highlighted by several key observations: (1) mast cell density in healthy myocardium is markedly lower compared to that of other immune cell types; (2) activation of mast cells triggers the release of a diverse array of mediators from their granules and induces de novo synthesis of further factors; (3) the effects of these mediators extend to various myocardial cell types, including cardiomyocytes, cardiac fibroblasts, cardiac endothelial cells, and other immune cells within the myocardium; (4) proteases released by mast cells play a substantial role in the activation of extracellular proteins and enzymes. 

**Figure 5:**
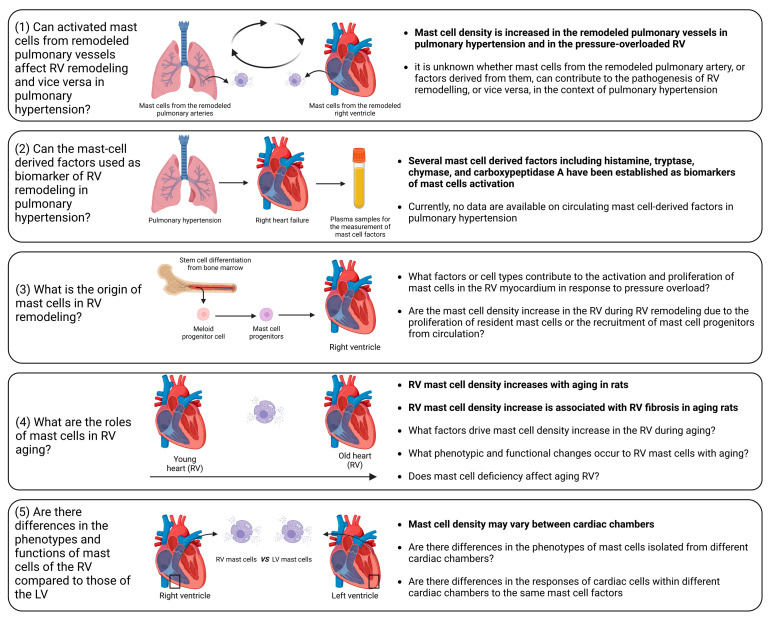
Unaddressed issues related to the role of mast cells in right ventricular remodeling. (1) Influence of activated mast cells from remodeled pulmonary vessels on RV remodeling and vice versa in pulmonary hypertension; (2) Circulating mast cell-derived factors as a biomarker of RV remodeling in pulmonary hypertension; (3) Origin of mast cells in remodeled RV; (4) Role of mast cells in RV aging; (5) Phenotypic and functional differences between mast cells in the RV and those in the left ventricle in health and disease. The known facts are highlighted in bold. The figure was created using BioRender.com, accessed on 25 November 2023.

Experimental evidence clearly showed that mast cell deficiency is associated with mitigation of the extent of cardiac injury and remodeling. Moreover, pharmacological inhibition of mast cell activation demonstrated its ability to alleviate adverse cardiac remodeling. It is important to emphasize that most investigations were devoted to the role of mast cells in left ventricular remodeling. Consequently, our understanding of the specific roles of mast cells in RV remodeling remains insufficiently explored (Figure 5).

Despite recent significant advances, there are still unaddressed issues related to the precise role of mast cells in RV remodeling and dysfunction, including the following (Figure 5):

(1) Influence of activated mast cells from remodeled pulmonary vessels on RV remodeling and vice versa in pulmonary hypertension. It is conceivable that various factors released by activated mast cells in remodeled pulmonary arteries may be released into the circulation and transported to the RV myocardium. These factors can potentially alter the responses of the RV to pressure overload. Additionally, there is evidence of increased mast cell activation in remodeled pulmonary arteries in pulmonary hypertension [193,194,195]. Similarly, it can be postulated that the release of various factors from activated mast cells within the RV myocardium may reach the pulmonary vasculature, where they could exacerbate the remodeling processes.
(2) Circulating mast-cell-derived factors as biomarkers of RV remodeling. Mediators released by activated mast cells, including histamine, tryptase, chymase, and carboxypeptidase A, can be measured in the systemic circulation and have the potential to serve as markers of mast cell activation in a number of conditions [196,197].
(3) Origin of mast cells in the remodeled RV. It remains to be elucidated whether the increase in mast cell density is caused by the proliferation of resident mast cells or the recruitment of mast cell progenitors from the circulation. To address this issue, reconstitution experiments with bone marrow-derived mast cells in mast cell-deficient mice subjected to PAB can be performed.
(4) Role of mast cells in RV aging. A correlation between age-related myocardial fibrosis and the density of mast cells has been previously revealed. However, there are still unanswered questions regarding mast cell-derived factors that define RV myocardial fibrosis during aging. Furthermore, which specific factors drive the increase in mast cell density in the RV during aging and what are the associated phenotypic and functional changes in RV mast cells? What are the consequences of mast cell deficiency on healthy RV aging? To date, it remains uncertain whether mast cell-deficient animals (mice or rats) maintain healthy aging of the RV.
(5) What factors govern mast cell activation and proliferation in the context of RV remodeling? Although pressure overload is the main cause of mast cell activation and proliferation during cardiac remodeling, identifying the key factors that regulate this process could offer a means for scientists to prevent the onset of mast cell activation by inhibiting upstream triggers. Mast cell activation may occur due to direct mechanical strain, as previous research has indicated mast cells sense the mechanical properties of their microenvironment [198]. Another possible scenario is that cardiomyocytes may release specific mediators during the initial phases in response to pathological stimuli, promoting mast cell activation and growth. Ultimately, exploring this issue could bring us closer to understanding the mechanisms behind the disease and developing pharmacological treatments.
(6) Differential impact of mast cell-derived factors on the RV and left ventricle. It is unclear which of the factors released by mast cells might have RV-specific effects in comparison to the left ventricle. It is unclear whether the effects of mast cell-derived factors differ between cardiac chambers due to compartment-specific differences in mast cell phenotypes or due to the chamber-specific phenotypes of cardiac cells. These differences in the cells targeted by mast cell factors might be partially explained by variations in receptor density or in the activated signaling pathways.

In summary, mast cells represent pivotal cellular entities implicated in diverse cardiac pathologies. The multifaceted roles and functions of mast cells in the RV remain incompletely explored, emphasizing the necessity of future studies with a dedicated focus on dissecting the contribution of mast cells to RV remodeling. 

## Figures and Tables

**Table 1:** Key features of mast cell-deficient mouse lines exploited in animal models of left and right ventricular remodeling.

Mouse Lines	Generation and Genetic Characteristics	Off-Target Phenotypes	LV Remodeling Models	RV Remodeling Models
Kit^W/W-v^	Compound heterozygotes, which carry two naturally occurring mutations (W and W-v) at the white spotting (W) locus [70] The W mutation is a point mutation at a splicing site in the transcript, resulting in the loss of the transmembrane domain, leading to impaired cell surface expression of KIT [79] The W-v mutation is a missense mutation within the KIT tyrosine kinase domain resulting in a significantly diminished kinase activity [70]	Altered intestinal intraepithelial lymphocyte homeostasis [80] Lack of interstitial cells of Cajal [81] High incidence of spontaneous dermatitis [82] Severe anemia [66] Infertility [66]	LAD-occlusion-induced ischemia-reperfusion cardiomyopathy [83] LAD-occlusion-induced myocardial infarction [84] Abdominal aorta banding-induced LV remodeling [85] TAC-induced atrial fibrillation [86]	PAB-induced RV remodeling [87]
Kit^W-sh/W-sh^	A spontaneous inversion mutation in the transcriptional regulatory elements upstream of the c-KIT transcription start site on mouse chromosome 5 [74]	Neutrophilia, megakaryocytosis, and thrombocytosis associated with splenomegaly and histological aberrations of the spleen [75] Deregulation of 27 genes located in the inverted genomic region, including disruption of corin, which causes cardiac hypertrophy [75] Impaired bone repair [88]	AngII-infusion model of LV remodeling [89] TAC-induced LV failure [90]	Not assessed
Cpa3^Cre/+^	Knock-in mice expressing Cre under control of the endogenous promoter of Cpa3 [91] Cpa3Cre/+ heterozygotes exhibit mast cell deficiency through Cre-mediated genotoxicity [91]	60% reduction in spleen basophils numbers [91]	LAD-occlusion-induced myocardial infarction [51]	Not assessed
mMCP4 knockout	Targeted inactivation of mMCP-4 gene [92]	Increased tryptase activity in peritoneal mast cells [93]	LAD-occlusion-induced myocardial infarction [94,95,96]	Not assessed

LV—left ventricle, RV—right ventricle, LAD—left anterior descending artery, PAB—pulmonary artery banding, AngII—angiotensin II, TAC—transverse aortic constriction, Cre—Cre recombinase, Cpa3—carboxypeptidase A3, mouse mast cell protease-4—mMCP-4.

**Table 2:** Mast cell numbers in the left and right ventricles.

Animal Model	Mast Cells in the RV	Mast Cells in the LV	Main Conclusion	References
Healthy C57BL/6J mice	Mast cell density 2.1 ± 0.25 cells/mm^2^	Mast cell density 1.09 ± 0.09 cells/mm^2^	Mast cell density is significantly higher in the RV as compared to the LV	Ingason et al. [103]
Healthy mongrel dogs	Mast cell density 6.53 ± 1.04 cells/mm^2^	Mast cell density 7.82 ± 1.16 cells/mm^2^	Mast cells are equally distributed between ventricles	Frangogiannis et al. [108]
Healthy young (6-month-old) and aging (12-month-old) Wistar rats	Significant increase in mast cell density in 12-month-old rats compared to 6-month-old rats Lower mast cell density in the RV than in the LV	Significant increase in mast cell density in the LV of 12-month-old rats compared to 6-month-old rats Greater mast cell density in the LV than in the RV	Increase in mast cell density in the myocardium with aging	Stamenov et al. [105]
Wistar rats (3-month-old) raised at sea level or simulated high altitude (3500 m)	No significant effect of altitude on mast cell density	Higher mast cell density in high altitude rats	Higher mast cell density in the RV than in the LV both at sea level and high altitude	Rakusan et al. [107]
Acute LAD occlusion in Sprague Dawley rats (2, 5, and 10 min)	Increase in RV histamine concentration after 2 min of LAD occlusion No change in the RV mast cell density after LAD occlusion No effects of mast cell stabilization	Decrease in the LV histamine concentration after 2 min of LAD occlusion No change in the LV mast cell density after LAD occlusion No effects of mast cell stabilization	Changes in myocardial histamine concentrations during acute myocardial ischemia are not related to mast cells	Dai et al. [109]
LAD occlusion (1 h) induced ischemia-reperfusion cardiomyopathy in rats	No significant changes in mast cell density following LAD	Increase in mast cell density in the infarct region of the LV at 1 day and 21 days after MI induction	Cardioprotective role of mast cell granules in MI via the prolonged survival of cardiomyocytes and the induction of angiogenesis	Kwon et al. [110]
Normotensive Wistar-Kyoto rats and SHR	Higher mast cell density in SHR	Higher mast cell density in SHR	Higher mast cell density in SHR and in the LV than in the RV independent of strain	Panizo et al. [106]
SHR with established hypertension and cardiac hypertrophy (6-month-old) and advanced or late-stage hypertension and cardiac hypertrophy (12-month-old)	Higher mast cell density in 12-month-old than 6-month-old SHR	Higher mast cell density in 12-month-old than 6-month-old SHR	Lower mean values for mast cell markers in the RV than the LV, irrespective of the age group of SHR	Kotov et al. [111]
Heart tissues from donor hearts and from patients with end-stage cardiomyopathy at the time of LVAD implantation and at the time of LVAD removal	No differences in mast cell density in RV compared to the LV	Higher mast cell density in cardiomyopathy than in donor hearts and lower than in LVAD-supported hearts Significant correlation between mast cell density and collagen in patients before LVAD implantation	Increase in mast cell density in cardiomyopathy A secondary increase in mast cell density due to mechanical support with LVAD and decrease in myocardial fibrosis	Akgul et al. [112]

LV—left ventricle, RV—right ventricle, LAD—left anterior descending artery, MI—myocardial infarction, SHR—spontaneously hypertensive rats, LVAD—left ventricular assist device.

## Data Availability

Not applicable.
